# Metformin has heterogeneous effects on model organism lifespans and is beneficial when started at an early age in *Caenorhabditis elegans*: A systematic review and meta‐analysis

**DOI:** 10.1111/acel.13733

**Published:** 2022-10-25

**Authors:** Austin J. Parish, William R. Swindell

**Affiliations:** ^1^ Meta‐Research Innovation Center at Stanford (METRICS) Stanford University Stanford California USA; ^2^ Department of Emergency Medicine, Chinle Comprehensive Health Care Facility Indian Health Services Chinle Arizona USA; ^3^ Department of Internal Medicine University of Texas Southwestern Medical Center Dallas Texas USA

**Keywords:** aging, lifespan, metformin, mice, nematodes, senescence, survival

## Abstract

There is growing interest in the use of metformin to extend lifespan and prevent the onset of age‐related disorders in non‐diabetic individuals. The impact of metformin on lifespan and aging has been studied in several model organisms, with varying effects. We conducted a systematic review of studies that performed laboratory experiments investigating the effect of metformin on overall lifespan in healthy *Mus musculus* mice and in *Caenorhabditis elegans* nematodes. Lifespan results for mice and nematodes were analyzed in separate meta‐analyses, and there was a significant amount of heterogeneity across experiments within each species. We found that metformin was not significantly associated with an overall lifespan‐prolonging effect in either mice or nematodes. For nematodes, however, there was a lifespan‐prolonging effect in experiments using live *OP50 Escherichia coli* as a food source, an effect that was larger when metformin was started earlier in life. Our work highlights the importance of testing compounds in a diversity of model organisms. Moreover, in all species, including humans, it may be necessary to study the effect of metformin on aging in both younger and older cohorts.

## Graphical abstract

Across studies, metformin seems to prolong lifespan in nematodes fed a specific bacterial diet but not in mice and may have a larger effect if started early in life.

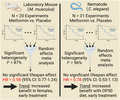

**Abbreviations**
AFT: accelerated failure time
CAMARADES: Collaborative Approach to Meta‐Analysis and Review of Animal Data in Experimental Stroke
C. elegans: Caenorhabditis elegans
DL: DerSimonian‐Laird
FUdR: fluorodeoxyuridine
HR: hazard ratio
PH: proportional hazards
SJ: Sidik‐Jonkman
TAME: Targeting Aging with Metformin
TJL: Jackson laboratory
UM: University of Michigan laboratory
UT: University of Texas laboratory

## 1 INTRODUCTION

The biguanide metformin has been used for years as a first‐line treatment for type 2 diabetes mellitus. However, there is growing interest in the potential use of metformin to prevent age‐related disease in individuals without diabetes (Barzilai et al., 2016; Kulkarni et al., 2020). The rationale for this is supported by prior work showing that metformin can prevent diabetes (Knowler, 2002) and various age‐related cancers (Bodmer et al., 2010; Landman et al., 2010). Further proof‐of‐principle was recently suggested by clinical trials showing that other diabetes medications, semaglutide and tirzepatide, can augment weight loss in overweight adults without diabetes (Jastreboff, 2022; Wilding, 2021). No randomized placebo‐controlled trial has supported metformin's use as a pro‐longevity intervention in normoglycemic humans, although epidemiological studies have suggested that metformin may prolong lifespan and have beneficial impacts on health beyond its antihyperglycemic effect (Campbell et al., 2017). Ultimately, definitive evidence regarding potential benefits of metformin in those without diabetes or prediabetes may be obtained from the Targeting Aging with Metformin trial (TAME) (Barzilai et al., 2016), which has been approved by the Food and Drug Administration and is among the first trials to focus on aging as a composite endpoint rather than a single disease‐oriented outcome.

The effects of metformin on longevity are challenging to study in human trials, and in prior work (including TAME), the only practical option has been to focus on biomarkers or composite endpoints as proxies for longevity and aging. On the contrary, mean and maximum lifespan have frequently been measured under controlled laboratory conditions in model organisms, such as the mouse and nematode. Studies in these organisms have demonstrated effects of metformin on multiple biochemical pathways relevant to aging (Kulkarni et al., 2020), showing that metformin activates AMPK and inhibits mTORC1 (Howell et al., 2017; Kalender et al., 2010; Martin‐Montalvo et al., 2013), activates SIRT1 (Cuyàs et al., 2018), decreases IGF‐1 levels (Sarfstein et al., 2013), reduces inflammation, attenuates cellular senescence (Moiseeva et al., 2013), enhances autophagy (Bharath et al., 2020), and reduces formation of reactive oxygen species by inhibiting mitochondrial complex I (Algire et al., 2012). Nevertheless, the effects of metformin on lifespan have varied among studies, with some showing a significant favorable effect (Anisimov et al., 2005), some showing no significant effect (Palliyaguru et al., 2020), and some showing a significant negative effect (Zhu et al., 2021).

The effects of an anti‐aging intervention on lifespan in model organisms can vary considerably between experiments, even though experiments are generally performed in controlled laboratory settings. The effects of dietary restriction in mice, for example, are highly sensitive to strain, with more robust and favorable effects seen in hybrid mice compared to inbred mice (Swindell, 2012), and overall diminution of favorable effects in wild‐derived mice (Harper et al., 2006). Likewise, the effects of rapamycin on lifespan in mice, although apparently more robust than those of caloric restriction, are more favorable in females compared to males (Swindell, 2017). These patterns have been discernable through meta‐analyses that compare findings across laboratories. Such work, for instance, has been performed to better understand the effects of rapamycin on laboratory mouse lifespan (Swindell, 2017) and the effect of resveratrol on longevity across many species, including mice and nematodes (Hector et al., 2012). To our knowledge, however, no prior meta‐analyses have been performed to evaluate the effects of metformin on lifespan in model organisms.

In this study, we aimed to quantify the impact of metformin on lifespan in the laboratory mouse *Mus musculus* (Kunstyr & Leuenberger, 1975) and the nematode *Caenorhabditis elegans* (*C. elegans*) (Olsen et al., 2006). We performed a systematic review and meta‐analysis of controlled experiments in which metformin was given to laboratory mice or to *C. elegans* and survival data was reported. We sought to systematically integrate studies to estimate overall effect sizes, determine if significant heterogeneity was present among experiments, and to identify experimental factors associated with such heterogeneity.

## 2 METHODS

### 2.1 Overall design of systematic review

This review was carried out following the PRISMA 2020 checklist for systematic reviews and meta‐analyses (Page et al., 2021) and previously published recommendations for systematic reviews of preclinical studies (Sena et al., 2014). The quality of studies was assessed using a modified version of the Collaborative Approach to Meta‐Analysis and Review of Animal Data in Experimental Stroke (CAMARADES) checklist (Macleod et al., 2004; Vesterinen et al., 2014). This study was prospectively registered with the PROSPERO database (https://www.crd.york.ac.uk/prospero/). This study was not funded. The authors have no competing interests. All data are available from the corresponding author (AJP) upon request.

### 2.2 Search and study extraction

We systematically searched PubMed, Web of Science, and Google Scholar for articles examining the impact of metformin on lifespan in model organisms; the specific search strategies used are summarized in Appendix 1. We also hand‐searched references of all included articles to find additional studies. The final search was performed on August 1, 2022. Both authors screened each abstract. Articles were excluded if they did not report novel experimental results with survival data (either summary statistics such as hazard ratios (HRs) or median lifespans, raw survival curves, or both); were studies in humans or organisms other than mice and *C. elegans*; used disease model mice (such as stroke models, see e.g., Winkler et al., 2001); administered metformin to mice via injection instead of oral intake; or were repeats of already included studies.

For each study, both authors in parallel extracted study characteristics including year of publication, specific mouse or *C. elegans* variant used, age metformin treatment was started, method of metformin delivery, and any special experimental characteristics. For mouse studies, we recorded the sex of mice used for each experiment and noted whether the strain was inbred or non‐inbred (hybrid/outbred). For nematode studies, we extracted the strain of *Escherichia coli* used for feeding in each experiment and whether it was alive or UV treated. We also extracted whether 5′‐fluorodeoxyuridine (FUdR) was used to prevent reproduction in these experiments (Wang et al., 2019). For each study, we extracted any reported lifespan data, including median or mean lifespans and Cox proportional HRs. In addition to summary data, we extracted any raw survival data; if Kaplan–Meier survival curves were available, they were converted to x‐y data using Plot Digitizer (http://plotdigitizer.sourceforge.net/) which was then transformed into survival data using R code (Wei & Royston, 2017). Additionally, the authors of all included studies were contacted and asked to provide raw survival data; whenever such data was made available, it was used for all calculations.

### 2.3 Outcomes

The primary outcome was the Cox proportional hazards (PH) HR (Bender et al., 2005). We also calculated as a secondary outcome the accelerated failure time (AFT) deceleration factor (percent increase or decrease in survivorship) using the Weibull distribution (Swindell, 2009). For each experiment, appropriateness of the Cox PH model was assessed using proportional hazard tests and log–log plots (Grambsh & Therneau, 1994). For both Cox PH and AFT models, censoring information was incorporated whenever available. All survival calculations were made using the survival package in R (Therneau, 2022). To check the accuracy of our algorithm for converting published survival curves to hazard ratio data, we used the simsurv package in R (Brilleman et al., 2021) to generate random survival datasets and each author independently estimated hazard ratio data from these datasets; these results are included in Table S1.

### 2.4 Meta‐analysis methods

Given a large amount of expected heterogeneity and the goal to generalize results beyond the specific experiments included in the meta‐analysis, we utilized random effects meta‐analysis. Random effects meta‐analysis was carried out using the Sidik‐Jonkman (SJ) estimator which has lower type I error rates than the DerSimonian‐Laird (DL) estimator (IntHout et al., 2014). Both included outcomes (hazard ratios and deceleration factors) were log‐transformed before meta‐analysis. Heterogeneity was estimated with the Higgins and Thompson's I^2^ statistic and Cochran's Q test (Sedgwick, 2012; von Hippel, 2015). Meta‐analysis calculations were performed using the meta package in R (Balduzzi et al., 2019). The possibility of publication bias or small study effects (Lin et al., 2020) was assessed using funnel plots and the Egger test for funnel plot asymmetry (Sterne et al., 2011); funnel plot analysis was performed using the metafor package in R (Viechtbauer, 2010).

One mouse study and four nematode studies contained two or more experiments that used a shared control group, yielding non‐independent effect size estimates. For the mouse study, the experiment with the larger sample size was included in calculations; for the nematode studies, as all experiments had similar sample sizes, the experiment with the most frequently used metformin dose across studies (50 mM) was used for calculations. For funnel plot analyses, in studies with multiple experiments, the single experiment with the largest sample size was selected as representative of the study. For all statistical tests, a nominal type I error rate was set at *α* = 0.05. The Benjamini–Hochberg method was used to adjust for multiple hypothesis testing across meta‐analysis subgroups (Benjamini & Hochberg, 1995). Version 4.0.0 of the R programming language was used for all calculations (R Core Team, 2020).

## 3 RESULTS

Our search strategy yielded 1068 studies. Of these, 446 (41.8%) were cancer studies, 167 (15.6%) were human studies, 163 (15.3%) were cell studies, 120 (11.2%) were reviews or commentaries without new data, 92 (8.6%) did not report any survival data related to aging, 28 (2.6%) were excluded disease models, 9 (0.9%) were repeats of other already included studies, and 8 (0.7%) exposed animals to both metformin and another molecule in the experimental group. Of the remaining 33 studies, 14 were excluded since they involved other organisms, and one was excluded since it was a mouse study that used subcutaneous injection of metformin. This left 10 mouse studies and 10 *C. elegans* studies. The PRISMA flow diagram is shown in Figure S1.

From the 10 mouse studies, we extracted 20 independent lifespan experiments, and from the 10 *C. elegans* studies, we extracted 31 independent lifespan experiments. The baseline properties of the studies and lifespan experiments are summarized in Table S2 for mice and Table S3 for *C. elegans*. Of the 10 mouse studies, one author provided survival data (Strong et al., 2016); of the 10 *C. elegans* studies, two authors provided survival data (Cabreiro et al., 2013; Wu et al., 2016).

### 3.1 Dosing and administration of metformin

For mice, we defined a “high dose” of metformin as an effective weight‐based dose of 500 mg/kg or higher; this was used in 3 of 20 experiments (15%). Mice received metformin either via drinking water (*n* = 10, 50%) or mixed in with chow (*n* = 10, 50%). In most experiments, mice received metformin daily (17 experiments, 85%). Alternate dosing schedules included giving metformin every other week daily (1 experiment), 5 days per week with 2 days off (1 experiment), and 5 days per month with the remaining days off (1 experiment).

We defined “early” administration of metformin as metformin dosing starting before 12 weeks (84 days) for mice; in turn, we defined early as dosing starting before Day 5 of adulthood for nematodes. These values were chosen based upon the distribution of start times among the available studies for each species, with the chosen times corresponding to gaps in the ordered sequence of start times. For mice, 6/20 (30%) experiments used early administration; for nematodes, 25/31 (81%) experiments used early administration.

For nematode experiments, 19 used OP50 *E. coli* as a food source (61%), three used HT115 *E. coli* (10%), and the remaining nine experiments (29%) used a variety of other *E. coli* strains (BL21G, CS180, CS2429, GD1, HB101, OP50‐MR, or OP50‐R26), *Bacillus subtilis*, or did not have a bacterial food source (axenic culture) (Lenaerts et al., 2008; Reinke et al., 2010). Of the 31 experiments, 19 used FUdR to prevent reproduction (61%).

### 3.2 Overall and subgroup analyses for studies in mice

**FIGURE 1:**
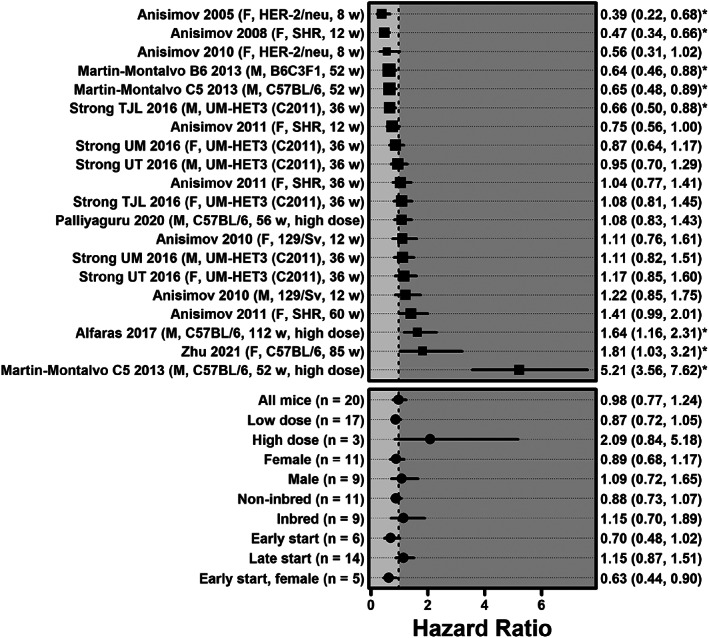
Forest plot of hazard ratios for all mouse experiments, as well as specific subgroups. The description on the left includes the primary author and publication year of the study including the experiment, as well as the sex, genotype, and age in weeks at which metformin was first administered. For the NIA ITP data from (Strong et al., 2016) the study site is also included (TJL = Jackson laboratory, UM = University of Michigan, UT = University of Texas). HRs and 95% CIs are listed on the right (* indicates *p* < 0.05). Larger square symbols are used for experiments with larger sample sizes. The meta‐analysis summary effects for all experiments (*n* = 20) as well as for specific subgroups are shown at the bottom.

Across all mouse experiments, metformin was not associated with a statistically significant reduction in hazard of death (HR = 0.98, 0.77–1.24, *p* = 0.84, *n* = 20), an effect with significant heterogeneity (*I* ^2^ = 87%, 82%–91%, *p* < 0.0001) (Figure 1). There was no significant effect of metformin on lifespan in several subgroups, including early start (HR = 0.70, 0.48–1.02, *p* = 0.32, *n* = 6), low dose experiments (HR = 0.87, 0.72–1.05, *p* = 0.40, *n* = 17), experiments with non‐inbred mice (HR = 0.88, 0.73–1.07, *p* = 0.40, *n* = 11) or experiments with only male mice (HR = 1.09, 0.72–1.65, *p* = 0.76, *n* = 9) or only female mice (HR = 0.89, 0.68–1.17, *p* = 0.58, *n* = 11). Metformin also did not significantly prolong lifespan in early start experiments with female mice (HR = 0.63, 0.44–0.90, *p* = 0.12, *n* = 5).

In univariate meta‐regression, there was no statistically significant difference between mice that received low dose metformin and mice that received high dose metformin (HR = 0.87 vs. 1.86, meta‐regression *p* = 0.056). There was no significant difference in survival according to start time (early HR = 0.70 vs. 1.15, meta‐regression *p* = 0.13), gender (male HR 0.89 vs. 1.09, meta‐regression *p* = 0.40), or genotype (non‐inbred HR = 0.88 vs. 1.15, meta‐regression *p* = 0.36). In bivariate meta‐regression controlling for early start and gender or early start and dose, early start did not have a significant effect on survival (*p* = 0.17, *p* = 0.17); however, when controlling for early start and genotype, early start was associated with improved survival (*p* = 0.042). Notably, none of the above results changed significantly after removing the Martin‐Montalvo 2013 high dose experiment (see Table S4). Mirroring hazard ratio results, metformin was not associated with a statistically significant AFT deceleration factor across all experiments (*D* = 1.01, 0.97–1.05, *p* = 0.66, *n* = 20), in early start experiments (*D* = 1.08, 0.98–1.18, *p* = 0.35, *n* = 6), low dose experiments (*D* = 1.03, 0.99–1.07, *p* = 0.36, *n* = 17), experiments with non‐inbred mice (*D* = 1.04, 0.98–1.09, *p* = 0.38, *n* = 11), male mouse experiments (*D* = 0.99, 0.94–1.05, *p* = 0.65, *n* = 9) female mouse experiments (HR = 1.03, 0.98–1.09, *p* = 0.48, *n* = 11), or early start experiments with female mice (*D* = 1.11, 1.01–1.21, *p* = 0.22, *n* = 5).

### 3.3 Overall and subgroup analyses for studies in nematodes

**FIGURE 2:**
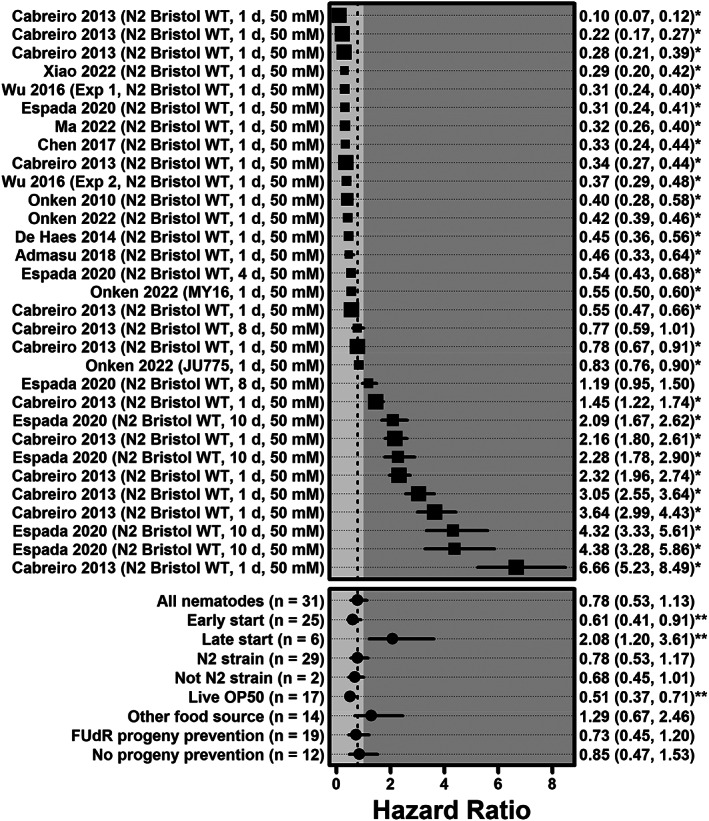
Forest plot of hazard ratios for all *Caenorhabditis elegans* experiments and specific subgroups. The description on the left includes the primary author and publication year of the study including the experiment, as well as the strain, age in days at which metformin was first administered, and the metformin dose. HRs and 95% CIs are listed on the right (* indicates *p* < 0.05). Larger square symbols are used for experiments with larger sample sizes. Meta‐analysis summary effects for all experiments (*n* = 31) as well as for specific subgroups are shown at the bottom (** indicates *p* < 0.05 after the Benjamini–Hochberg correction for multiple comparisons).

Across all *C. elegans* experiments, metformin was not associated with a statistically significant reduction in hazard of death (HR = 0.78, 0.53–1.13, *p* = 0.19, *n* = 31), an effect with significant heterogeneity (*I* ^2^ = 96%, 95%–97%, *p* < 0.0001) (Figure 2). Early start experiments showed a significant reduction in mortality (HR = 0.61, 0.41–0.91, *p* = 0.045, *n* = 25), while late start experiments were associated with increased mortality (HR = 2.08, 1.20–3.61, *p* = 0.041, *n* = 6).

Early start of metformin administration was associated with significantly improved survival compared with later start (HR = 0.61 vs. 2.08, meta‐regression *p* = 0.017). There was no significant difference between experiments using N2 strains and other strains (HR = 0.78 vs. 0.68, meta‐regression *p* = 0.85), or between experiments that used FUdR for progeny prevention and those that did not (HR = 0.73 vs. 0.85, meta‐regression *p* = 0.85). However, experiments using live OP50 *E. coli* were associated with improved survival compared with those using other food sources (HR = 0.51 vs. 1.29, meta‐regression *p* = 0.017).

For nematodes, metformin was not associated with a significant AFT deceleration factor across all experiments (*D* = 1.07, 0.98–1.17, *p* = 0.15, *n* = 31), or in early start experiments (*D* = 1.13, 1.02–1.25, *p* = 0.057, *n* = 25), or late start experiments (*D* = 0.85, 0.74–0.97, *p* = 0.057). Metformin was associated with a significant deceleration factor in experiments using live OP50 *E. coli* (*D* = 1.20, 1.10–1.31, *p* = 0.0009, *n* = 17).

For nematodes, there was a significant, inverse dose–response relationship between HR and day of treatment start, with each additional day increasing the log hazard ratio by 0.15 (*p* = 0.0034); this relationship remained after adjusting for strain, food source and use of FUdR (*β* = 0.15, *p* = 0.0099). For mice, there was no significant relationship between HR and week of treatment start (*β* = 0.003, *p* = 0.88), including after adjusting for dose, sex, and strain (*β* = −0.011, *p* = 0.64).

### 3.4 Study quality and publication bias

The quality of studies varied, with a median CAMARADES quality sum of 4 (IQR: 3–5) for mouse studies and 3 (IQR: 2–3) for nematode studies, out of a total of 9 (see Figure S2). Of note, no mouse study described blinding, and only one (10%) described allocation concealment. Similarly, only two nematode studies (20%) described blinding, and none described allocation concealment. Three of the 10 mouse studies (30%) described sample size calculations, and only one of the 10 nematode studies (10%) did so.

**FIGURE 3:**
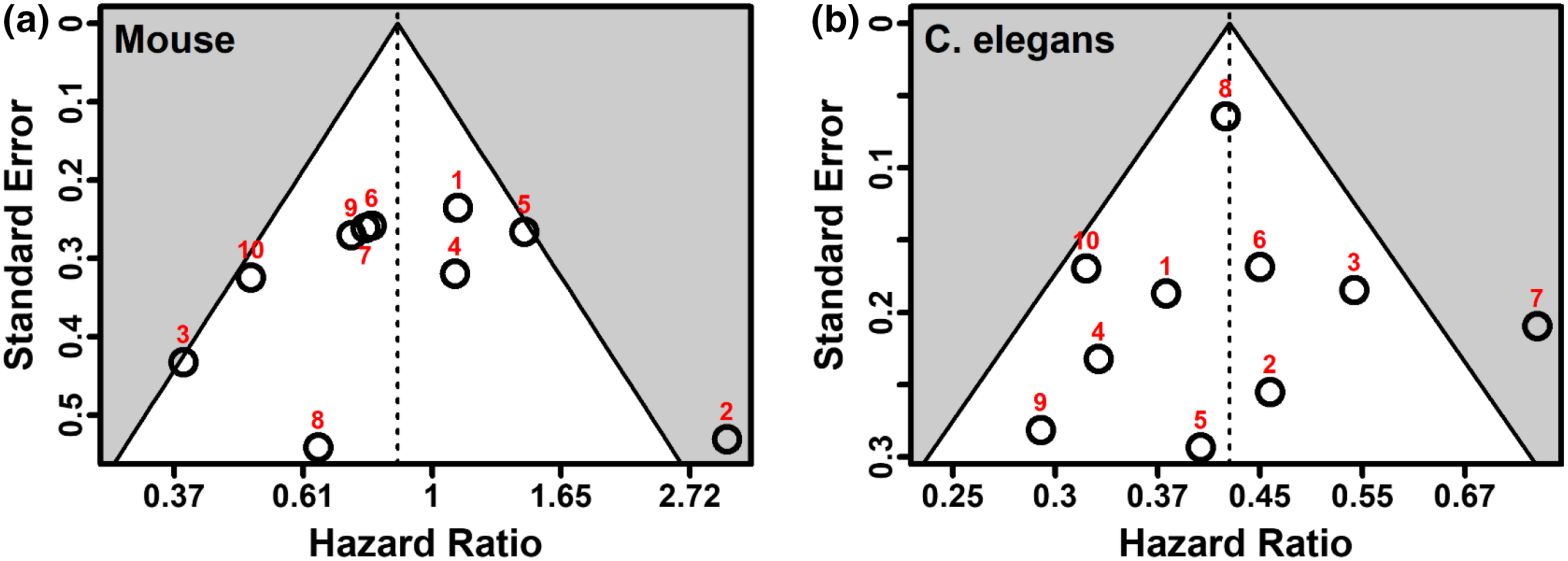
*Left*: Funnel plot for 10 mouse studies; Egger's *p* = 0.92. *Right*: Funnel plot for 10 *Caenorhabditis elegans* studies; Egger's *p* = 0.98. Both plots show standard error versus log (hazard ratio) for the largest experiment from that publication; vertical lines show the pooled random effects estimate for each set of experiments (HR = 0.81 for mice, 0.42 for nematodes). Including all 20 mouse experiments resulted in an Egger's *p* = 0.72; including all 31 *C. elegans* experiments resulted in an Egger's *p* = 0.59. For the list of associated studies, see Tables S5a,b.

Using funnel plots and Egger's test for funnel plot asymmetry, we did not find significant evidence of publication bias; these results are summarized in Figure 3 below.

## 4 DISCUSSION

Studies of purported anti‐aging molecules in model organisms provide important data for delineating mechanisms of activity and assessing translatability to humans. In addition to growing epidemiological evidence, a variety of preclinical studies in model organisms have connected metformin to beneficial effects in both healthspan and lifespan (Cabreiro et al., 2013; Crimmins, 2015; Piskovatska et al., 2019). The anti‐aging mechanisms of metformin are still being deciphered, but there are likely diverse pathways involved, with differential effects among model organisms. In this systematic review and meta‐analysis, we found that metformin has heterogenous effects on lifespan within species as well as differing effects between mice and nematodes.

There was no significant association between metformin supplementation and lifespan in mice; in subgroup analyses, we found no significant difference in outcomes between experiments with male and female mice or between inbred and non‐inbred mouse strains. Prior published studies are also non‐definitive regarding sex‐specific effects of metformin on mouse longevity, with some studies demonstrating benefit in only female mice (Anisimov et al., 2010) and others demonstrating benefit in only males (Zhu et al., 2021). In humans, metformin was reported to lower fasting plasma glucose and 2‐h postprandial glucose more in females compared to males (Li et al., 2021), and one large‐scale study showed that female diabetics treated with metformin had a lower incidence of cardiovascular events than male diabetics (Raparelli et al., 2020). Further studies will be needed to understand whether sex moderates the effect of metformin on mouse longevity. Overall, the longevity results reported here for metformin differ from previous results for rapamycin in mice (Swindell, 2017), which was found to robustly decrease mortality across experiments. Similar to rapamycin, we found a larger survival benefit of metformin when started early in life or when administered to female mice, however, neither of these associations were statistically significant in the current study.

Higher metformin doses in mice were associated with less survival benefit, potentially due to toxic effects, such as lactic acidosis, vitamin B12 deficiency, or nephrotoxicity (Alfaras et al., 2017). Unfortunately, it is difficult to separate the impact of higher doses from later start times, as all the high dose experiments included in this review also had a late start time for metformin supplementation. Notably, redoing the analyses after removing all high dose experiments did not significantly change our results. In humans, however, metformin is contraindicated when eGFR declines below 30, and some loss of renal function is expected with age in mice, particularly inbred as compared with non‐inbred mice (Hackbarth & Harrison, 1982). It is thus possible that attenuation of metformin benefits at higher doses and later start times reflects emergent metformin toxicity secondary to age‐related renal decline.

In contrast to our findings in mice, across several experiments and subgroups metformin had a significant effect on the lifespan of *C. elegans*. This disparity may be due to the significant differences in biology between the two organisms. For example, metformin may uniquely impact *C. elegans* lifespan via alteration of *E. coli* metabolism, a central food source for laboratory‐maintained *C. elegans*, leading to downstream methionine restriction; notably, metformin was found to reduce lifespan in *C. elegans* grown on axenic cultures without *E. coli* (Cabreiro et al., 2013). Food source significantly altered the effect of metformin on *C. elegans* lifespan in our meta‐analysis, although the majority of experiments using a food source other than live OP50 *E. coli* came from one study (Cabreiro et al., 2013). Espada et al. (2020) used UV treated OP50 and HT115 *E. coli* but only with metformin started late in life. Although metformin may alter the intestinal microbiome (Brunkwall & Orho‐Melander, 2017), such effects of metformin on methionine metabolism may be less prominent in mice. Future studies of metformin's effect in mice with or without a methionine‐restricted diet may be worthwhile to evaluate whether dietary methionine is required for positive effects on mouse lifespan.

Early administration of metformin was associated with a significantly larger beneficial effect on lifespan for nematodes; but this effect was not statistically significant for mice. Espada et al. (2020) found metformin to be harmful if started late in the nematode lifespan, finding that although metformin activated specific longevity promoting pathways in young nematodes it led to ATP exhaustion and cell death in older nematodes. Possible mechanisms for an age‐dependent effect of metformin on lifespan in mice have yet to be elucidated. Importantly, none of the studies included in this review included experiments where metformin was started in both young and old cohorts of mice under otherwise similar experimental conditions. However, a two‐by‐two factorial experiment to evaluate the effects of start time (early vs. late) and sex (male vs. female), and the interaction between these factors, would be feasible to perform in mice. Our results highlight this experiment as an important direction for future work. Additionally, further studies of metformin's effect on the lifespan of *C. elegans* to investigate the interaction between food source and start time will be important.

Although the overall absence of a significant effect on lifespan in mice compared with *C. elegans* on an OP50 diet may be due to biological differences, it also possible that in mice, healthspan may be increased without a significant impact on lifespan due to competing risks (Garmany et al., 2021; Koller et al., 2012). Indeed, certain studies included in this review which did not show a significant prolongation of lifespan nevertheless found improved insulin sensitivity, reduced hepatic steatosis, and other beneficial health effects in mice (Alfaras et al., 2017). Similarly, a study in Fischer‐344 rats found metformin to have beneficial effects on body weight and adipose tissue, without any appreciable impact on lifespan (Smith et al., 2010). Additionally, even if metformin has lifespan‐prolonging effects, such effects may be smaller than healthspan effects and require larger sample sizes to detect (Richardson et al., 2016).

### 4.1 Limitations

Our study has several limitations. First, we were only able to obtain original survival curve or hazard ratio data from the authors of one of 10 mouse studies and two of 10 nematode studies. For the remainder, the survival data were reconstructed from the published survival curves, which invariably introduces noise into effect size estimates. To mitigate this, effect size estimates were produced by both authors separately and averaged; the estimates produced by both authors aligned closely, differing by 5% or less for most experiments.

Second, by only including three databases we may have systematically missed unpublished or non‐English language data, although we attempted to reduce this risk by searching gray literature (via Google Scholar) as well as manual searches of references. In our analysis, we did not find significant evidence of small study effects through funnel plot analysis, although the power of these tests is limited given the small number of included studies (Sterne et al., 2011).

Notably, survival curves of 7 of 20 mouse experiments (35%) and 15 of 31 nematode experiments (48%) showed evidence of nonproportional hazards, determined via visual inspection of log–log plots. However, given the similar trends obtained from the meta‐analyses of deceleration factors, it is unlikely that nonproportionality significantly biased outcomes.

Finally, the identified studies for both mice and nematodes were generally of low quality, with almost none reporting methods of allocation concealment or blinding and less than a quarter reporting sample size calculations; only half reported randomization methods. The lack of these design features may have biased the reported effects (Hirst et al., 2014).

## 5 CONCLUSION

There is increasing interest in the possible use of metformin to increase healthspan and prevent onset of age‐related disorders in non‐diabetic individuals. In this systematic review and meta‐analysis, we found metformin to have no significant overall effect on the lifespan of healthy mice, nor any significant beneficial effect in studied subgroups. In contrast, we found significant evidence of lifespan prolonging effects of metformin in *C. elegans* nematodes fed a diet of OP50 *E. coli* and starting metformin supplementation earlier in the nematode lifespan led to more beneficial effects on lifespan. Important next steps for preclinical research into metformin's potential role as a geroprotector would include mouse experiments of daily metformin supplementation started at different points in the natural lifespan. Such studies can be formulated as factorial designs to also study the interaction between metformin start time and sex, which should yield insights into the relationship between these two factors and associated mechanisms. A greater understanding of this could in turn have important implications for clinical research in humans and may indicate a need for trials to be run in both younger and older cohorts.

## AUTHOR CONTRIBUTIONS

Austin J. Parish conceived and designed analysis, collected data, performed analysis, and wrote and edited manuscript. William R. Swindell collected data, performed analysis, and wrote and edited manuscript.

## CONFLICT OF INTEREST

The authors have no conflicts of interest to disclose.

## Supporting information


Appendix S1

Appendix S2

## Data Availability

All data utilized in this study will be made publicly available upon publication via the Open Science Framework (osf.io).

## APPENDIX 1

### SEARCH STRATEGIES USED IN THIS STUDY

**PubMed Search Strategy**

(metformi*[Title] OR biguanid*[Title] OR “N‐N‐dimethylbiguanide”[Title] OR glucophage[Title]) AND ((lifespan[Title] OR “life span”[Title] OR “life‐span”[Title] OR survival[Title] OR healthspan[Title/Abstract] OR “health‐span”[Title/Abstract] OR longevity[Title/Abstract] OR aging[Title/Abstract] OR ageing[Title/Abstract]) OR aging[majr])

**Web of Science Search Strategy**

TI = ((metform* OR biguanid* OR “N‐N‐dimethylbiguanide” OR glucophag*) AND (lifespan OR “life span” OR “life‐span” OR survival OR healthspan OR “health‐span” OR longevity OR aging OR ageing OR senescen*)).

**Google Scholar Search Strategy**

allintitle: metformin (lifespan OR “life span” OR “life‐span” OR longevity OR aging OR ageing OR healthspan OR “health span” OR “health‐span” OR “survival”).
