# Supplementary material for: Metformin has heterogeneous effects on model organism lifespans and is beneficial when started at an early age in Caenorhabditis elegans: A systematic review and meta‐analysis
Source: Aging Cell. 2022 Oct 25;21(12):e13733. doi: 10.1111/acel.13733 (PMC9741508; doi:10.1111/acel.13733)

**Supplementary Material**

**Supplementary Table 1.** Results of applying authors’ algorithm for generating hazard ratios from survival data to 10 simulated survival datasets.

| Curve | Actual HR | Author 1 HR | Author 1 HR Absolute % Error | Author 2 HR | Author 2 HR Absolute % Error |
| --- | --- | --- | --- | --- | --- |
| 1 | 0.512 | 0.500 | 2.3 | 0.510 | 0.46 |
| 2 | 0.612 | 0.597 | 2.5 | 0.605 | 1.20 |
| 3 | 0.661 | 0.678 | 2.6 | 0.661 | 0.01 |
| 4 | 0.871 | 0.847 | 2.8 | 0.854 | 2.0 |
| 5 | 0.732 | 0.714 | 2.5 | 0.732 | 0.02 |
| 6 | 0.766 | 0.759 | 0.9 | 0.774 | 1.0 |
| 7 | 0.855 | 0.865 | 1.2 | 0.882 | 3.2 |
| 8 | 0.381 | 0.370 | 2.9 | 0.391 | 2.5 |
| 9 | 0.677 | 0.689 | 1.8 | 0.679 | 0.30 |
| 10 | 0.927 | 0.939 | 1.3 | 0.939 | 1.30 |
|  |  | *Average Absolute % Error* | 2.1 |  | 1.2 |

**Supplementary Table 2.** Characteristics of twenty mouse metformin lifespan experiments included in this review, from ten published studies.

| **First author and year** | **Experiment ID** | **Variant ( inbred or non-inbred)** | **Number of mice** | **Gender of mice** | **Age that metformin was started at (weeks)** | **Metformin vehicle** | **Metformin dose** | **Other experimental characteristics** |
| --- | --- | --- | --- | --- | --- | --- | --- | --- |
| Palliyaguru 2020 | 1 | C57BL/6 (Inbred) | 99 | Male | 56 | Chow | 1% (10 g/kg chow) | High fat diet |
| Zhu 2021 | 2 | C57BL/6 (Inbred) | 52 | Female | 85 | Drinking water | 100 mg/kg |  |
| Anisimov 2005 | 3 | HER-2/neu (Inbred) | 66 | Female | 8 | Drinking water | 100 mg/kg | HER-2 neu transgenic |
| Anisimov 2010 | 4 | 129/Sv (Inbred) | 96 | Female | 12 | Drinking water | 100 mg/kg |  |
|  | 5 | 129/Sv (Inbred) | 87 | Male | 12 | Drinking water | 100 mg/kg |  |
| Alfaras 2017 | 6 | C57BL/6 (Inbred) | 102 | Male | 112 | Drinking water | 1% |  |
| Anisimov 2011 | 7 | SHR (Outbred) | 170 | Female | 12 | Drinking water | 100 mg/kg |  |
|  | 8 | SHR (Outbred) | 142 | Female | 36 | Drinking water | 100 mg/kg |  |
|  | 9 | SHR (Outbred) | 102 | Female | 60 | Drinking water | 100 mg/kg |  |
| Strong 2016 | 10 | UM-HET3 (C2011) (Hybrid) | 154 | Male | 36 | Chow | 0.1% w/w | National Institute on Aging Interventions testing program |
|  | 11 | UM-HET3 (C2011) (Hybrid) | 144 | Female | 36 | Chow | 0.1% w/w |  |
|  | 12 | UM-HET3 (C2011) (Hybrid) | 147 | Male | 36 | Chow | 0.1% w/w |  |
|  | 13 | UM-HET3 (C2011) (Hybrid) | 143 | Female | 36 | Chow | 0.1% w/w |  |
|  | 14 | UM-HET3 (C2011) (Hybrid) | 141 | Male | 36 | Chow | 0.1% w/w |  |
|  | 15 | UM-HET3 (C2011) (Hybrid) | 134 | Female | 36 | Chow | 0.1% w/w |  |
| Anisimov 2010 | 16 | HER-2/neu (Inbred) | 35 | Female | 8 | Drinking water | 100 mg/kg |  |
| Martin-Montalvo 2013 | 17 | B6C3F1 (Hybrid) | 333 | Male | 52 | Chow | 0.1% w/w |  |
|  | 18 | C57BL/6 (Inbred) | 235 | Male | 52 | Chow | 0.1% w/w |  |
|  | 19 | C57BL/6 (Inbred) | 90 | Male | 52 | Chow | 1% w/w |  |
| Anisimov 2008 | 20 | SHR (Outbred) | 100 | Female | 12 | Drinking water | 100 mg/kg in drinking water |  |

**Supplementary Table 3**. Characteristics of 31 *C. elegans* metformin lifespan experiments included in this review, from ten published studies. All experiments used a metformin dose of 50 mM.

| **First author and year** | **Experiment ID** | ***C. elegans* variant** | **Bacterial food source** | **Number of nematodes in experimental group** | **Age that metformin was started at (days)** | **FUdR used for progeny prevention** |
| --- | --- | --- | --- | --- | --- | --- |
| Admasu 2018 | 1 | N2 Bristol WT | OP50 (live) | 75 | 1 | No |
| Cabreiro 2013 | 2 | N2 Bristol WT | CS180 (live) | 269 | 1 | Yes |
|  | 3 | N2 Bristol WT | OP50-R26 (live) | 255 | 1 | Yes |
|  | 4 | N2 Bristol WT | OP50 (live) | 105 | 1 | Yes |
|  | 5 | N2 Bristol WT | GD1 (live) | 149 | 1 | Yes |
|  | 6 | N2 Bristol WT | Bacillus subtilis (live) | 284 | 1 | Yes |
|  | 7 | N2 Bristol WT | OP50 (live) | 101 | 8 | Yes |
|  | 8 | N2 Bristol WT | HB101 (live) | 339 | 1 | Yes |
|  | 9 | N2 Bristol WT | CS2429 (live) | 249 | 1 | Yes |
|  | 10 | N2 Bristol WT | OP50 (UV treated) | 238 | 1 | Yes |
|  | 11 | N2 Bristol WT | HT115 (live) | 283 | 1 | Yes |
|  | 12 | N2 Bristol WT | BL21G (live) | 247 | 1 | Yes |
|  | 13 | N2 Bristol WT | OP50-MR (live) | 280 | 1 | Yes |
|  | 14 | N2 Bristol WT | Axenic | 288 | 1 | Yes |
| Chen 2017 | 15 | N2 Bristol WT | OP50 (live) | 100 | 1 | Yes |
| De Haes 2014 | 16 | N2 Bristol WT | OP50 (live) | 169 | 1 | No |
| Espada 2020 | 17 | N2 Bristol WT | OP50 (live) | 140 | 1 | No |
|  | 18 | N2 Bristol WT | OP50 (live) | 142 | 4 | No |
|  | 19 | N2 Bristol WT | OP50 (live) | 140 | 8 | No |
|  | 20 | N2 Bristol WT | OP50 (UV treated) | 140 | 10 | No |
|  | 21 | N2 Bristol WT | HT115 (live) | 143 | 10 | No |
|  | 22 | N2 Bristol WT | HT115 (UV treated) | 141 | 10 | No |
|  | 23 | N2 Bristol WT | OP50 (live) | 140 | 10 | No |
| Ma 2022 | 24 | N2 Bristol WT | OP50 (live) | 200 | 1 | No |
| Onken 2010 | 25 | N2 Bristol WT | OP50 (live) | 711 | 1 | No |
| Onken 2022 | 26 | N2 Bristol WT | OP50 (live) | 150 | 1 | Yes |
|  | 27 | MY16 | OP50 (live) | 150 | 1 | Yes |
|  | 28 | JU775 | OP50 (live) | 150 | 1 | Yes |
| Wu 2016 | 29 | N2 Bristol WT | OP50 (live) | 158 | 1 | Yes |
|  | 30 | N2 Bristol WT | OP50 (live) | 165 | 1 | Yes |
| Xiao 2022 | 31 | N2 Bristol WT | OP50 (live) | 88 | 1 | No |

**Supplementary Table 4.** Sensitivity experiment showing hazard ratio results after removing the Martin-Montalvo 2013 high dose experiment.

|  | *Sensitivity Experiment* |
| --- | --- |
| **Analysis (n)** | **Hazard Ratio Result (95% CI, p-value)** |
| All mouse experiments (n = 19) | 0.92 (0.76 to 1.11, 0.62) |
| Early start (n = 6) | 0.70 (0.48 to 1.02, 0.32) |
| Late start (n = 13) | 1.01 (0.85 to 1.21, 0.90) |
| Low dose (n = 17) | 0.87 (0.72 to 1.05, 0.40) |
| High dose (n = 2) | 1.31 (0.89 to 1.93, 0.40) |
| Inbred mice (n = 8) | 0.96 (0.66 to 1.38, 0.90) |
| Non-inbred mice (n = 11) | 0.88 (0.73 to 1.07, 0.40) |
| Male mice (n = 8) | 0.93 (0.73 to 1.20, 0.75) |
| Female mice (n = 11) | 0.90 (0.68 to 1.19, 0.64) |
| Early start and female mice (n = 5) | 0.63 (0.44 to 0.90, 0.12) |

**Supplementary Table 5a.** Ten mouse lifespan studies included in the funnel plot for Figure 4a.

| **Study Number** | **Study Name** | **Study First Author and Year** |
| --- | --- | --- |
| 1 | Combining a high dose of metformin with the SIRT1 activator, SRT1720, reduces lifespan in aged mice fed a high-fat diet | Palliyaguru 2020 |
| 2 | Effect of Metformin on Cardiac Metabolism and Longevity in Aged Female Mice | Zhu 2021 |
| 3 | Effect of metformin on life span and on the development of spontaneous mammary tumors in HER-2/neu transgenic mice | Anisimov 2005 |
| 4 | Gender differences in metformin effect on aging, life span and spontaneous tumorigenesis in 129/Sv mice | Anisimov 2010 |
| 5 | Health benefits of late-onset metformin treatment every other week in mice | Alfaras 2017 |
| 6 | If started early in life, metformin treatment increases life span and postpones tumors in female SHR mice | Anisimov 2011 |
| 7 | Longer lifespan in male mice treated with a weakly estrogenic agonist, an antioxidant, an ?-glucosidase inhibitor or a Nrf2-inducer | Strong 2016 |
| 8 | Metformin extends life span of HER-2/neu transgenic mice and in combination with melatonin inhibits growth of transplantable tumors in vivo | Anisimov 2010 |
| 9 | Metformin improves healthspan and lifespan in mice | Martin-Montalvo 2013 |
| 10 | Metformin slows down aging and extends life span of female SHR mice | Anisimov 2008 |

**Supplementary Table 5b.** Ten *C. elegans* lifespan studies included in the funnel plot for Figure 4b.

| **Study Number** | **Study Name** | **Study First Author Year** |
| --- | --- | --- |
| 1 | An Ancient, Unified Mechanism for Metformin Growth Inhibition in C. elegans and Cancer | Wu 2016 |
| 2 | Drug Synergy Slows Aging and Improves Healthspan through IGF and SREBP Lipid Signaling | Admasu 2018 |
| 3 | Late life metformin treatment limits cell survival and shortens lifespan by triggering an aging-associated failure of energy metabolism | Espada 2020 |
| 4 | Metformin extends C. elegans lifespan through lysosomal pathway | Chen 2017 |
| 5 | Metformin induces a dietary restriction-like state and the oxidative stress response to extend C. elegans Healthspan via AMPK, LKB1, and SKN-1 | Onken 2010 |
| 6 | Metformin promotes lifespan through mitohormesis via the peroxiredoxin PRDX-2 | De Haes 2014 |
| 7 | Metformin retards aging in C. elegans by altering microbial folate and methionine metabolism | Cabreiro 2013 |
| 8 | Metformin treatment of diverse Caenorhabditis species reveals the importance of genetic background in longevity and healthspan extension outcomes | Onken 2022 |
| 9 | Metformin induces s-adenosylmethionine restriction to extend the Caenorhabditis elegans Healthspan through H3K4me3 modifiers | Xiao 2022 |
| 10 | Low-dose metformin targets the lysosomal AMPK pathway through PEN2 | Ma 2022 |

**Supplementary Figure 1.** PRISMA flow diagram for the review.


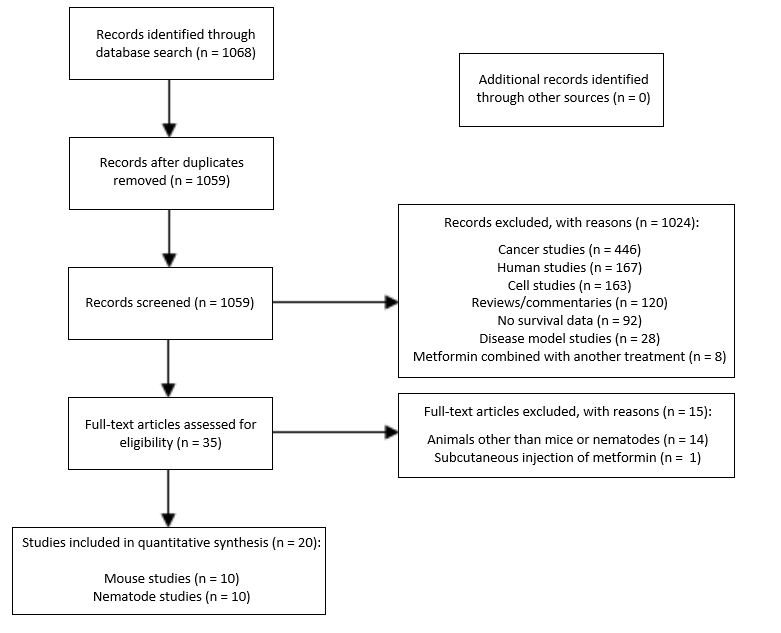


**Supplementary Figure 2.** CAMARADES quality scores for each included study, along with total quality score (out of a possible maximum of 9).


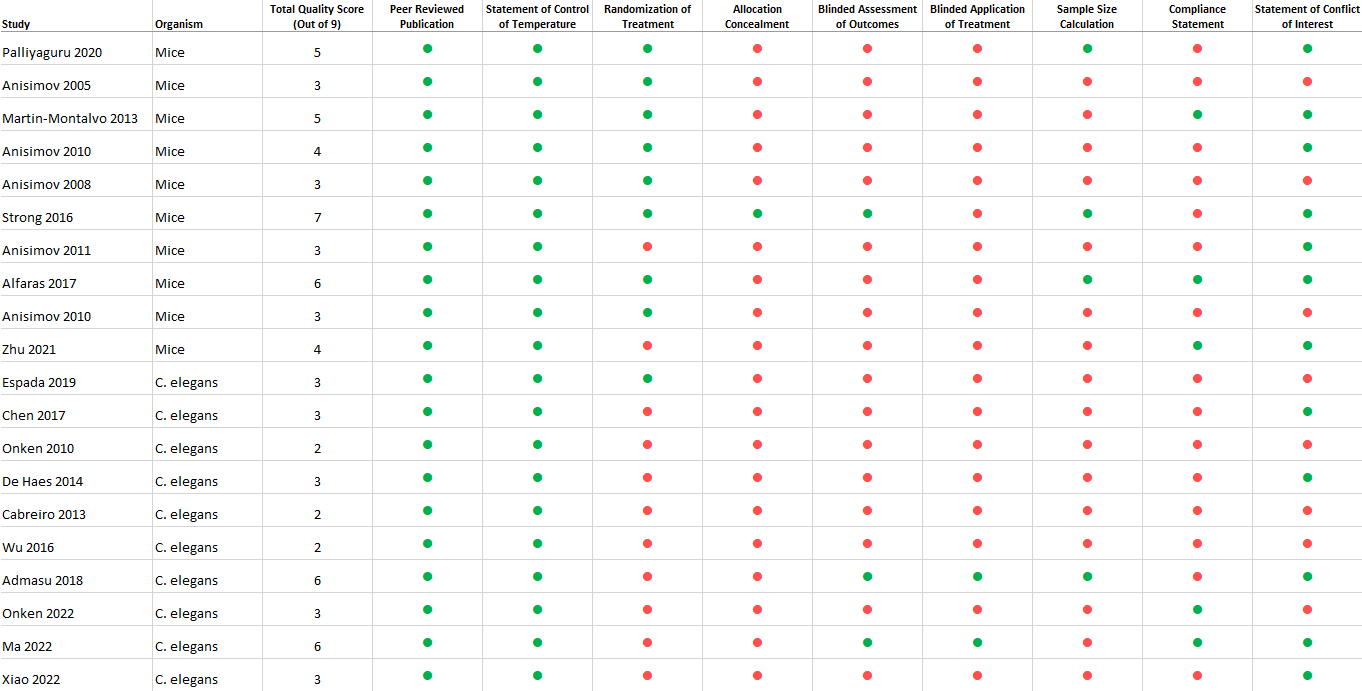

Supplement: Supplementary file 1 — Appendix S1 [file ACEL-21-e13733-s001.docx]
